# The clinicopathological significance of *NAB2‐STAT6* gene fusions in 52 cases of intrathoracic solitary fibrous tumors

**DOI:** 10.1002/cam4.572

**Published:** 2015-12-21

**Authors:** Shih‐Chiang Huang, Chien‐Feng Li, Yu‐Chien Kao, I‐Chieh Chuang, Hui‐Chun Tai, Jen‐Wei Tsai, Shih‐Chen Yu, Hsuan‐Ying Huang, Jui Lan, Shao‐Lun Yen, Po‐Chun Lin, Tse‐Ching Chen

**Affiliations:** ^1^ Department of PathologyChang Gung Memorial HospitalChang Gung University College of MedicineTaoyuanTaiwan; ^2^ Department of PathologyChi‐Mei Medical CenterTainanTaiwan; ^3^ Department of PathologyShuang Ho HospitalTaipei Medical UniversityTaipeiTaiwan; ^4^ Departments of PathologyKaohsiung Chang Gung Memorial Hospital and Chang Gung University College of MedicineKaohsiungTaiwan; ^5^ Department of PathologyChanghua Christian HospitalChanghuaTaiwan; ^6^ Department of Anatomic PathologyE‐Da HospitalKaohsiungTaiwan; ^7^ Departments of OrthopedicsKaohsiung Chang Gung Memorial Hospital and Chang Gung University College of MedicineKaohsiungTaiwan

**Keywords:** NAB2, solitary fibrous tumor, STAT6, thoracic, translocation

## Abstract

*NAB2‐STAT6* gene fusion drives STAT6 nuclear expression and is the pathognomonic hallmark of solitary fibrous tumors (SFTs). However, no study has systematically analyzed the clinicopathological features, STAT6 immunoexpression status, or the fusion variants of *NAB2‐STAT6* in intrathoracic SFTs. Fifty‐two intrathoracic SFTs were retrieved to appraise histopathology, assess STAT6 immunoexpression, and determine *NAB2‐STAT6* fusion variants by RT‐PCR. Location‐relevant histologic mimics served as controls. Thirty‐one pleura‐based, 12 mediastinal/pericardial, and nine intrapulmonary lesions were histologically categorized into eight malignant, eight atypical, and 36 conventional or cellular SFTs, including two fat‐forming and two giant cell angiofibroma‐like SFTs. STAT6 distinctively decorated the tumoral nuclei in 51 (98%) SFTs. However, no nuclear staining was observed in the histological mimics. *NAB2‐STAT6* fusion was detected in 34 SFTs. Twenty‐nine (85.3%) exhibited the major *NAB2ex4‐STAT6ex2/3* variant and 5 (14.7%) the minor *NAB2ex6‐STAT6ex16/17*. *NAB2ex4‐STAT6ex2* was significantly associated with older age (*P* = 0.01) and pleuropulmonary tumors (*P* = 0.025). After a median follow‐up of 33.9 (range, 0.3–174.6) months, adverse outcomes occurred in one atypical and five malignant SFTs, including two local relapses, one intrapulmonary metastasis, and three extrathoracic metastases. Inferior disease‐free survival was univariately associated with atypical/malignant histology (*P* = 0.001) and a mitosis >4/10 HPFs (*P* = 0.0012) but was unrelated to fusion variants. In conclusion, the majority of intrathoracic SFTs exhibited STAT6 nuclear staining, and *NAB2ex4‐STAT6ex2/3* was the predominant fusion type. However, clinical aggressiveness is associated with atypical/malignant histology primarily contributed by increased mitosis but was unrelated to the *NAB2‐STAT6* fusion variants.

## Introduction

A solitary fibrous tumor (SFT) is a rare fibroblastic tumor of intermediate malignant potential, which predominantly occurs in middle‐aged adults and prototypically arises from the thoracic cavity as a pleural tumor 1, 2. However, SFTs can be anatomically ubiquitous and involve the lung parenchyma, mediastinum, and a wide variety of extrapleural sites. Histologically, a SFT is characterized by proliferative ovoid to spindly tumor cells arranged in a patternless architecture with alternating cellularity and an elaborate vasculature of staghorn vessels. Some histologic variants, such as myxoid, giant cell angiofibroma‐like, and fat‐forming SFTs, have been reported 2. In the past, SFTs were principally diagnosed by a combined assessment of clinical context, morphological features, and CD34 immunohistochemistry with imperfect sensitivity and specificity 2, 3. Although most intrathoracic SFTs are clinically indolent, approximately 10–20% of cases may behave aggressively and develop recurrences or metastases years after a primary resection 2, 4, 5. However, it is challenging to predict the clinical outcome for individual cases.

Using next‐generation sequencing, an in‐frame fusion of the neighboring NGFI‐A binding protein 2 (*NAB2*) and signal transducer and activator of transcription 6, interleukin‐4 induced (*STAT6*) genes on chromosome 12q13 was recently identified to be the pathognomonic genetic event of SFTs 6, 7. The reported detection rates of *NAB2‐STAT6* gene fusion in SFT samples range from 55 to 100% 6, 7, 8, 9, 10, 11. A chimeric *NAB2‐STAT6* fusion transcript may exhibit highly variable breakpoints across exons from the 5′‐end of *NAB2* and 3′‐end of *STAT6*. Subsequently, different groups reported the diagnostic utility of nuclear immunoexpression of STAT6 as a surrogate marker of the *NAB2‐STAT6* fusion, which may facilitate the discrimination between SFTs and histological mimics 12, 13.

Intriguingly, a recent analysis of the *NAB2‐STAT6* exon composition revealed probable correlations of fusion variants with histopathological characteristics and biological behavior of SFTs arising from various sites 9. Specifically, the *NAB2ex4‐STAT6ex2/3* variant was preferentially detected in classic pleuropulmonary SFTs featuring extensive fibrosclerotic stroma and mostly indolent behavior. In contrast, the *NAB2ex6‐STAT6ex16/17* variant was more frequently associated with extrathoracic sites, increased cellularity, and clinical aggressiveness. Focusing on 52 intrathoracic SFTs, we aimed to robustly characterize the frequencies of various *NAB2‐STAT6* fusion types, evaluate the STAT6 nuclear immunoexpression, and appraise the possible impact of immunohistochemical and molecular findings on clinicopathological features and clinical aggressiveness.

## Materials and Methods

### Study cohort

This study was performed with the approval of the institutional review board. In this retrospective series, patients with intrathoracic tumors diagnosed as SFTs and resected between 2000 and 2014 were identified from the consultation file of one author (HYH) and pathological archives of Kaohsiung and Linkou Chang Gung Memorial Hospitals. A systematic histological reappraisal was conducted by participating pathologists using multi‐headed microscopy. The final study cohort comprised 52 SFTs. Based on the latest WHO Classification 2, these SFTs were histologically categorized as the conventional variant in 36 cases, atypical in 8, and malignant in 8. To designate malignant SFTs, it requires more than four mitoses per 10 high‐power fields (HPFs) with or without hypercellularity, nuclear pleomorphism, and infiltrative border. Atypical SFTs were defined by marked nuclear pleomorphism with limited mitotic activity ≤4/10 HPFs. Other histological variants were also evaluated, that is, lipomatous and giant cell angiofibroma‐like SFT variants. The medical charts were reviewed to ascertain clinical characteristics and the dates of local recurrences and metastasis.

### Immunohistochemistry

A representative formalin‐fixed, paraffin‐embedded (FFPE) tissue block from each of the 52 SFTs and 14 cases of histological mimics was re‐cut to perform STAT6 immunohistochemistry. The tissue sections were deparaffinized, rehydrated, and microwave‐heated for antigen retrieval using a routine protocol. The sections were then incubated with a monoclonal STAT6 antibody (1:100, YE361, GeneTex, Hsinchu City, Taiwan). Blind to clinicopathological data, one author (SCH) independently evaluated the slides and scored the labeling intensity of STAT6 as weak, moderate, or strong and the staining extent as 0 (negative), 1+ (1–25%), 2+ (26–50%), 3+ (51–75%), or 4+ (>75%) in the tumoral nuclei. Cytoplasmic staining was interpreted as negative.

### Molecular testing

There were 34 FFPE intrathoracic SFTs resected within 5 years. Three 10‐*μ*m‐thick tissue scrolls were cut for RNA extraction using RecoverAll Total Nucleic Acid isolation kit (Ambion Inc., Austin, TX). For two cases with available fresh tumor tissue, the total RNA was extracted by RNeasy Mini‐kit (Qiagen, Valencia, CA). The yield and quality of mRNA were determined by a NanoDrop UV spectrophotometer (Thermo Scientific, Wilmington, DE). The ImPromII Reverse Transcriptase (RT) System (Promega, Madison, WI) was used to synthesize the cDNA for a subsequent polymerase chain reaction (PCR).

Using 2.5 units of Platinum Taq DNA polymerase (Invitrogen, Waltham, MA), we performed the PCR with the above cDNA product. The primer pairs targeting the 5′ exons of *NAB2* and the 3′ exons of *STAT6* were newly designed based on various exon compositions reported in the literature and are listed in Table S1 7. The thermal conditions started with a denaturing heating at 95°C for 5 min, followed by an amplification of 35 (fresh tissue) or 38 (FFPE specimens) cycles, and a final elongation step at 72°C for 10 min. Specifically, the amplification cycles were 95°C for 30 sec, a touchdown gradient from 62 to 59°C for 30 sec each in cycles 1–4, annealing at 58°C for the remaining cycles, and extension at 72°C for 45 sec. The polymerase chain reaction products were examined on agarose gels and sequenced on an automated sequencer (Applied Biosystems 3730 DNA Analyzer, Life Technologies, Carlsbad, CA).

### Statistical analysis

Associations and comparisons of *NAB2‐STAT6* fusion variants or STAT6 immunoexpression with various clinicopathological parameters were evaluated using a Chi‐square or Fisher exact test for categorical variables and a Mann–Whitney test for continuous variables. The end point evaluated was disease‐free survival, which was defined as the time between the date of surgery and date of recurrence or metastasis. In a univariate survival analysis, Kaplan–Meier curves were plotted. The difference between the groups was compared by a log–rank test. For all analyses, two‐sided tests of significance were used with a *P* < 0.05 considered to be significant.

## Results

After the histological reappraisal of the 62 retrospectively retrieved cases, the original diagnosis of an intrathoracic SFT in the pathological reports were in doubt for 10 cases. As shown in Figure S1, these included four primary pleuropulmonary synovial sarcomas, one sarcomatoid mesothelioma, four sarcomatoid carcinomas of the lung, one mediastinal cellular SFT‐like dedifferentiated liposarcoma, one mediastinal spindle cell/sclerosing rhabdomyosarcoma, and one PEComa with prominent spindle cell histology. In the immunohistochemical evaluation of STAT6 expression and subcellular localization, these excluded mimics and two additional cases of each pulmonary sarcomatoid carcinoma and mesothelioma were used as the negative controls.

### Clinicopathological features of the intrathoracic SFTs

In the study cohort (Table 1), there were 22 men and 30 women with SFTs, ranging in age at operation from 30 to 85 years (median, 56; mean, 57.6). Thirty‐one SFTs were found to primarily originate in the pleura, 11 in the mediastinum, nine in the pulmonary parenchyma, and one in the pericardium. Of 50 cases with available information, tumor size ranged from 1.2 to 27.5 cm (median, 6.3; mean, 9.8) in the greatest dimension. Doege–Potter's syndrome was observed in one patient with a malignant pleural SFT who presented with synchronous paraneoplastic hypoglycemia.

**Table 1:** Clinicopathological and molecular features of 52 intrathoracic solitary fibrous tumors

	Case number (percentage)
Gender	52
Male	22 (42%)
Female	30 (58%)
Age (range)	52
<55	23 (44%)
≥55	29 (56%)
Locations	52
Pleura	31 (60%)
Lung	9 (17%)
Mediastinum/pericardium	12 (23%)
Size (cm)	50
<5 cm	17 (34%)
≥5 cm	33 (66%)
Histologic type	52
Conventional/cellular	36 (70%)
Atypical	8 (15%)
Malignant	8 (15%)
Mitotic figure (/10 HPF)	52
0	31 (60%)
1–4	13 (25%)
>4	8 (15%)
STAT6 immunostaining intensity	52
Negative	1 (2%)
Weak	1 (2%)
Moderate	5 (10%)
Strong	45 (86%)
STAT6 immunostaining extent	52
0 + (0%)	1 (2%)
1 + (1–24%)	0
2 + (25–49%)	4 (8%)
3 + (50–75%)	6 (12%)
4 + (76–100%)	41 (78%)
*NAB2‐STAT6* RT‐PCR assay	34/36
*NAB2ex4‐STAT6ex2/3* fusion variant	29 (85%)
*ex4‐ex2*	28 (82%)
*ex4‐NAB2(I)‐ex3* a	1 (3%)
*NAB2ex6‐STAT6ex16/17* fusion variant	5 (15%)
*ex6‐ex17* b	4 (12%)
*ex6‐ex16*	1 (3%)
Poor quality	2

a This case had an intact exon 4 followed by a short stretch (8 bps) of inserted *NAB2* intronic sequence and a cytosine of undetermined origin, which was then with truncated *STAT6* exon 3.

b One of these 4 solitary fibrous tumors harboring *NAB2ex6‐STAT6ex17* fusion showed incorporation of one undetermined cytosine and additional 18 nucleotides from the 16th intron of *STAT6* gene.

**Figure 1:**
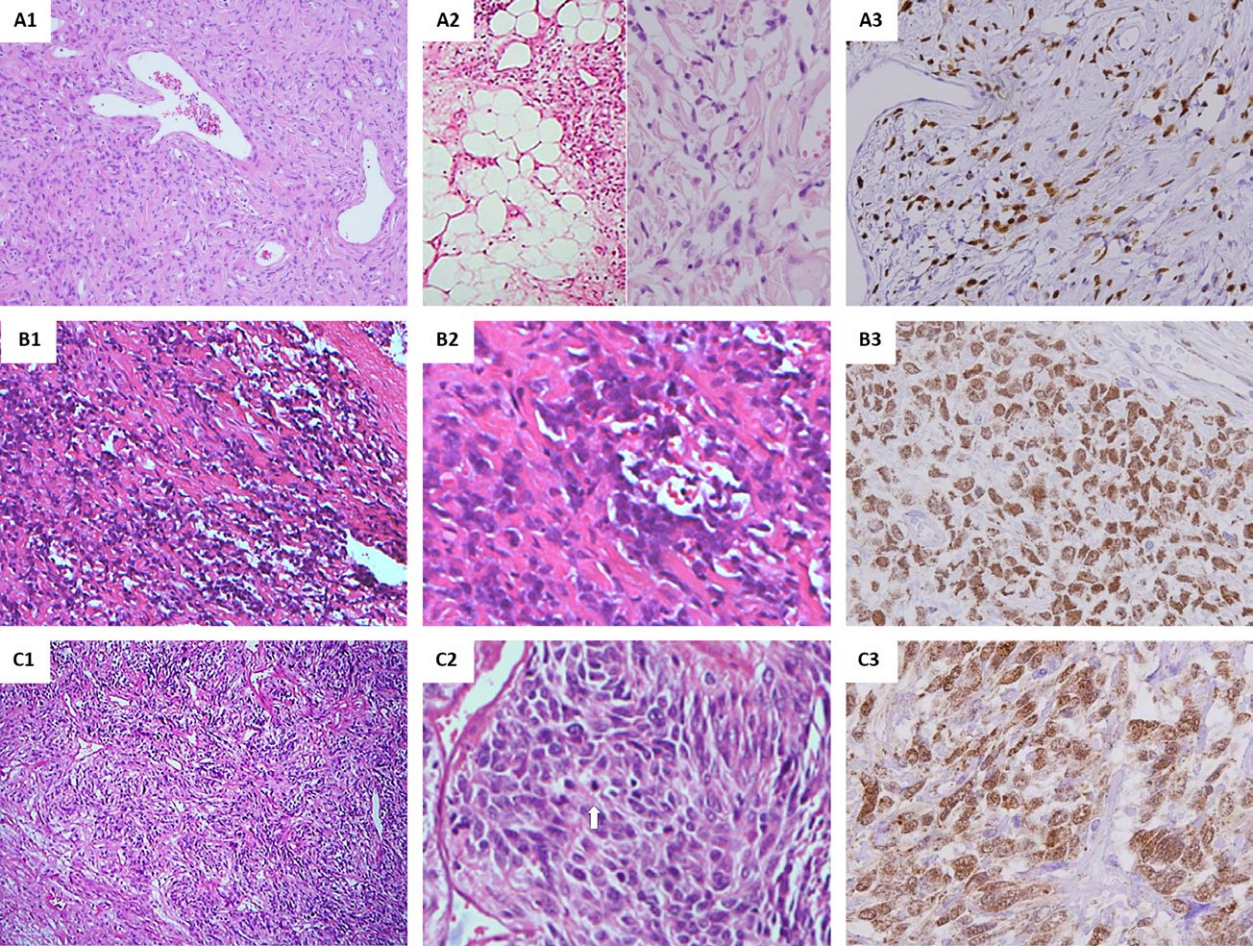
Histological features and nuclear expression of STAT6 in the different morphological variants of intrathoracic solitary fibrous tumors (SFTs). A conventional pleura‐based SFT harbored the predominant *NAB2ex4‐STAT6ex2* fusion variant (A1) and crisp STAT6 nuclear staining (A3). One mediastinal fat‐forming SFT (A2, left) and one SFT with giant cell angiofibroma‐like histology (A2, right) were illustrated. One pleural atypical SFT harboring *NAB2ex6‐STAT6ex17* showed a prominent nuclear pleomorphism (B1‐2) and STAT6 nuclear expression (B3). One pleura‐based malignant SFT with the *NAB2ex4‐STAT6ex2* fusion variant was characterized by hypercellular proliferation of atypical tumoral cells (C1) and apparently increased mitoses (C2). Nuclear STAT6 expression was diffuse (C3).

Histologically, 32 SFTs were classified as conventional and displayed characteristic haphazard storiform or fascicular growth of spindle cells in a predominantly hypocellular fibrocollagenous stroma (Fig. 1A_1_) having generally uniform, vesicular nuclei and low mitotic rates between 0 and 1 per 10 HPFs. The four cellular SFTs showed diffusely increased cellularity and were histologically equivalent to the so‐called hemangiopericytomas, two of which were focally fat‐forming with lobules of mature adipocytes (Fig. 1A_2_, left). Notably, two conventional SFTs focally presented with cracked pseudoangiomatous spaces surrounded by multinucleated stromal cells (Fig. 1A_2_, right) reminiscent of patterns of giant cell angiofibromas. There were eight atypical SFTs (Fig. 1B_1‐2_) that exhibited nuclear pleomorphism and a mitotic rate <4 per 10 HPFs without necrosis. Designated as malignant (Fig. 1C_1‐2_), eight SFTs exhibited diffusely increased cellularity and increased mitosis from 5 to 30 per 10 HPFs (mean, 11), with prominent nuclear pleomorphism seen in three cases and coagulative tumor necrosis in 1.

### STAT6 immunohistochemical analysis

There was no STAT6 nuclear staining in any control case (Fig. S1). In contrast, 51 of 52 intrathoracic SFTs (98%) showed positive nuclear STAT6 staining (Fig. 1), including all 34 cases with detectable *NAB2‐STAT6* transcripts (see below). Only one histologically conventional, pleura‐based tumor was STAT6‐negative (Table 1). The STAT6 nuclear staining intensity was strong in 45 cases, moderate in 5, and weak in 1, with the extent being interpreted as diffuse (≥50% of nuclei) in 47 cases and focal (<50% of nuclei) in 4. In all, the vast majority of SFTs (43, 82.7%) exhibited diffuse and strong nuclear reactivity.

### Detection of *NAB2‐STAT6* gene fusion variants

**Figure 2:**
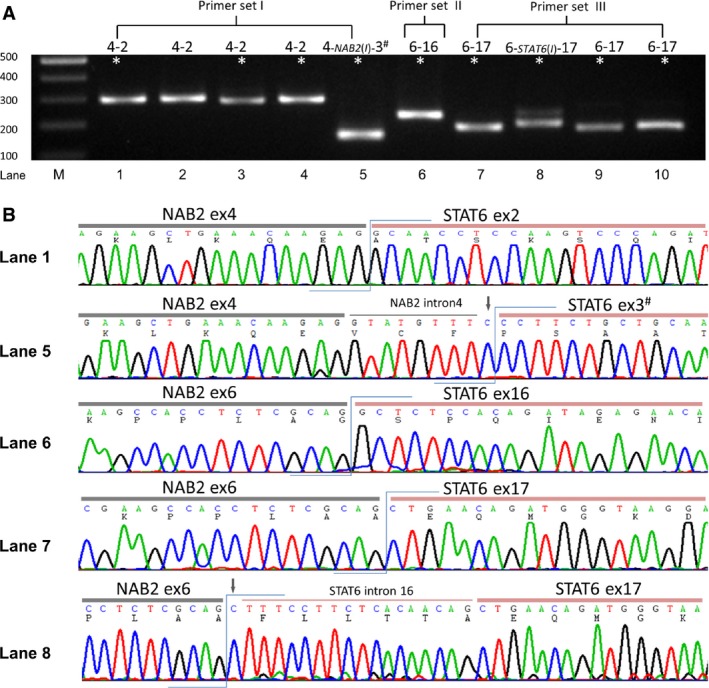
RT‐PCR assay for the *NAB2‐STAT6* fusion transcripts of the intrathoracic solitary fibrous tumors (SFTs). (A) PCR products of representative cases with 5 different exon composition types were detected by three primer pairs labeled as I‐III. The lane loaded with a 100‐bp DNA ladder marker was labeled as M, followed by Arabic numbers 1–10 for individual SFTs from left to right. The asterisked samples were validated by Sanger sequencing. (B) Partial sequencing chromatograms showed junctions of the *NAB2‐STAT6* chimeric transcripts in the representative cases. Note that the inserted *NAB2* and *STAT6* intronic sequences, as indicated by the thin overhead horizontal lines, were identified in the case with the *NAB2ex4‐STAT6ex3* variant (lane 5) and one of 4 cases with *NAB2ex6‐STAT6ex17* variant (lane 8), respectively. The downward arrows indicates nucleotides of undetermined origins between the exons and introns, including one cytosine behind the *NAB2* intron 4 (8 bp) and one cytosine located 5′ to the *STAT6* intron 16 (17 bp). ^#^, exon integrity interrupted by the junction breakpoints; *I, NAB2* or *STAT6* intron.

Of the 36 cases tested by RT‐PCR, various *NAB2‐STAT6* transcripts were successfully detected in two fresh and 32 formalin‐fixed samples (Fig. 2, Table 1). By Sanger sequencing (Fig. 2B), the most common *NAB2ex4‐STAT6ex2* variant was present in 28 cases, with the functionally equivalent *NAB2ex4‐STAT6ex3* product being detected in only one case. In the remaining five informative cases, the *NAB2ex6‐STAT6ex17* variant was detected in four cases, while the functionally equivalent *NAB2ex6‐STAT6ex16* variant was found in one case. At the mRNA level, all but two cases demonstrated breakpoints at the individual junctional 3′‐ends of *NAB2* exons and 5′‐ends of *STAT6* exons participating in the involved fusions without incorporated intronic sequences. Notably, one case had an intact exon 4 followed by a short stretch (8 bps) of inserted *NAB2* intronic sequence and a cytosine of undetermined origin, which was then with truncated *STAT6* exon 3. The other case featuring *NAB2ex6‐STAT6ex17* harbored one undetermined cytosine and 17 bps of *STAT6* intronic sequence before the starting nucleotide of the *STAT6* exon 17.

**Figure 3:**
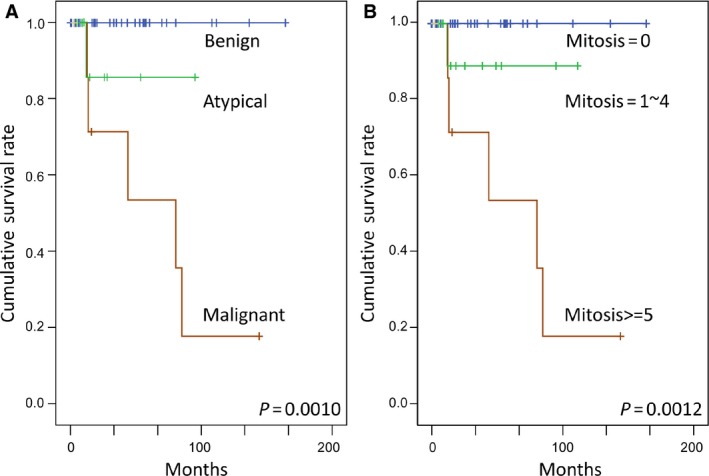
Log‐rank univariate survival analyses. Histological classification (A) and mitotic activity (B) were both significantly predictive of worse disease‐free survival.

### Correlations of the *NAB2‐STAT6* fusion variants with clinicopathological features and STAT6 immunoexpression

As shown in Table 2, we found that the patients with SFTs harboring the *NAB2ex4‐STAT6ex2/3* transcript were significantly older at presentation (*P* = 0.010), indicating an age‐related variability in the exon compositions of *NAB2‐STAT6* fusion transcripts. In fact, all 18 patients who had the major *NAB2ex4‐STAT6ex2/3* variant in their SFTs were 55 years or older. The five patients with SFTs harboring *NAB2ex6‐STAT6ex16/17* were all younger than 55 years. The *NAB2ex4‐STAT6ex2/3* variant was significantly predominant in the pleuropulmonary tumors. Albeit a small case number, the mediastinal or pericardial SFTs more commonly presented with the *NAB2ex6‐STAT6ex16/17* variant (*P* = 0.025). Along with one case with the minor fusion variant, all 29 SFTs with the major *NAB2*ex4*‐STAT6*ex2/3 fusion variant displayed diffuse STAT6 nuclear staining. It is intriguing that two of five cases harboring the minor *NAB2ex6‐STAT6ex16/17* fusion variant displayed only focal STAT6 nuclear expression (*P* < 0.001).

**Table 2:** Associations of *NAB2‐STAT6* gene fusion variants with clinicopathological parameters in intrathoracic solitary fibrous tumors

	Correlation with *NAB2‐STAT6* gene fusion				Disease‐free survival		
	Case No.	*NAB2‐STAT6* fusion		*P*‐value	Case No.	Event	*P*‐value
		*ex4‐Sex2/3*	*ex6‐ex16/17*				
Sex				0.841			0.0793
Male	19	13	2		29	2	
Female	15	16	3		21	4	
Age (years)				0.010			0.3401
<55	16	11	5		28	2	
≥55	18	18	0		22	4	
Location				0.025			0.2749
Pleura	25	23	2		30	4	
Lung	6	5	1		9	2	
Mediastinum/pericardium	3	1	2		11	0	
Histologic classification				0.348			0.0010
Conventional/cellular	21	18	3		32	0	
Atypical	6	6	0		10	1	
Malignant	7	5	2		8	5	
Tumor Size (cm)				0.307			0.8654
<5	13	12	1		14	1	
≥5	19	15	4		34	5	
Mitotic Count (/10HPF)				0.490			0.0012
0	20	18	2		30	0	
1–4	7	6	1		12	1	
>4	7	5	2		8	5	
*NAB2‐STAT6* fusion type							0.1781
*ex4‐ex2/3*	–	–	–		27	2	
*ex6‐ex16/17*	–	–	–		5	2	

### Survival analysis

Follow‐up data were available for 50 patients with a median duration of 33.9 months (mean, 50.5; range, 0.3–174.6). Forty‐four patients (88%) had no evidence of disease at the time of the last follow‐up, including seven atypical and three malignant SFTs. Adverse outcomes occurred in one of eight patients with atypical SFTs (12.5%) and five of eight malignant SFTs (62.5%), including local recurrence (2), intrapulmonary metastasis (1), and distant extrathoracic dissemination (3), each to the liver, adrenal, and humeral bone, respectively. Four patients (8%) died of disease, including one with atypical and three with malignant SFTs. As for the malignant SFT, the 5‐year and 10‐year disease‐free survival rates were 0.686 and 0.171, respectively, while the overall survival rate was 0.833 at 5 years and 0.278 at 10 years. As shown in Table 2, worse disease‐free survival was univariately associated with atypical/malignant histology (*P* = 0.001, Fig. 3A) and mitosis >4/10 HPFs (*P* = 0.0012, Fig. 3B). However, fusion variants were unrelated to disease‐free survival (*P* = 0.1781), despite a tendency of relatively indolent behavior in those with the *NAB2ex4‐STAT6ex2/3* fusions.

## Discussion

Although SFTs most commonly arise from the thorax, especially the pleura, they only account for less than 5% of all neoplastic diseases in this anatomical region 1. Intrathoracic SFTs are considered to be derived from pluripotent fibroblastic cells in the submesothelial connective tissue and topographically classified into pleural (visceral/parietal), mediastinal, and pulmonary origins 1. Intrapulmonary SFTs are assumed to develop from the visceral pleura or interlobular septa but intriguingly grow toward the lung parenchyma in an inverted pattern with clinical behavior akin to the pleura‐based SFTs 14. However, mediastinal SFTs tend to follow a more aggressive course due to their complicated location that may hamper complete surgical resection 1. Because a minority of intrathoracic SFTs may present with adverse outcomes in the long run, better identification of high‐risk cases remains a pivotal issue in SFT management. Indeed, imaging features and pathologic criteria have been individually or jointly used with varying usefulness to prognosticate the malignant potential 15, 16, 17, 18.

The diagnosis of malignant SFT is principally based on a pathologic evaluation of four essential histologic variables proposed by England and colleagues, including mitotic count >4/HPF, necrosis, hypercellularity, and nuclear atypia 15. A subsequent study further highlighted the prognostic utility of mitotic activity as well as the histologic appearance of frankly sarcomatous transformation 19. In our study, intrathoracic SFTs were categorized into the conventional, atypical, and malignant variants. There was a strong association between disease‐free survival and histologic classification. Although few cases with adverse events precluded the multivariate analysis, the prognostic impact of the histological classification could be mainly attributable to the contribution of increased mitotic activity because it showed almost the same statistical power as the histological classification. Recently, Tapias et al. proposed a new scoring system that integrates both macroscopic (e.g., pleural origin, gross appearance, and tumor size) and microscopic (e.g., hypercellularity, necrosis or hemorrhage, and mitotic figures) features, which was claimed to better predict the recurrence of resected pleural SFTs 18. Although the overall long‐term survival rate and disease‐free survival rate are generally worse in malignant SFTs, previously reported overall survival rates were better in cases undergoing a complete surgical resection. These results indicated that surgical resection remains the mainstay in the management of malignant SFTs 17, 20. Because adjuvant chemotherapy exhibits a degree of antitumor activity in advanced or metastatic SFTs, its efficacy needs further validation 21. Nevertheless, when completely resected, some histologically malignant SFTs may behave in an uneventful fashion and rare, bland‐appearing SFTs may develop late local recurrences or a malignant transformation. The uncertainty of a prognosis in a small subset of SFTs renders a long‐term follow‐up imperative for all patients.

With the discovery of the novel *NAB2*‐*STAT6* gene fusion in SFT 6, 7, several studies recently adopted next‐generation sequencing and/or RT‐PCR to report on exon compositions in different fusion variants 8, 9, 10, 11. As shown in Table 3, specifically for the intrathoracic SFTs, the compiled data from prior studies and our series demonstrated that the vast majority (83%) are the *NAB2ex4‐STAT6ex2/3* fusions, including *NAB2ex4‐STAT6ex2* and *NAB2ex4‐STAT6ex3* in 73 and 21 cases, respectively. However, the minor *NAB2ex6‐STAT6ex16/17* fusions only account for 7% of cases, including *NAB2ex6‐STAT6ex17* in 7 and *NAB2ex6‐STAT6ex16* in 1, which are then followed by miscellaneous *NAB2ex2‐STAT6ex2/6, NAB2ex4‐STAT6ex5/18, NAB2ex6‐STAT6ex18,* and *NAB2ex7‐STAT6ex3* fusions not detected in this study. Interestingly, as many as 90% of intrapulmonary SFTs demonstrate the *NAB2ex4‐STAT6ex2/3* fusion variant, further reinforcing the notion that intrapulmonary SFTs fall into the spectrum of pleural SFTs. Barthelmeß et al. 9 alluded to the possibly of indolent behavior of SFTs indicating *NAB2ex4‐STAT6ex2/3* fusions from various anatomic sites given their lower mitotic activity and cellularity. However, there was no survival analysis in their study comparing the prognostic impact between different fusion variants. In our series of intrathoracic SFTs, cases with the *NAB2ex4‐STAT6ex2/3* fusions were associated with older age, pleuropulmonary location, and a tendency for more diffuse STAT6 nuclear immunoexpression. The *NAB2*‐*STAT6* fusion patterns were found not to be prognostically significant.

**Table 3:** The summary of *NAB2*‐*STAT6* fusion patterns in intrathoracic solitary fibrous tumors

Series	Cases	*NAB2ex4‐STAT6ex2/3*	*NAB2ex6‐STAT6ex16/17*	Others	Negative
Chmielecki et al. 6	5	5 (100%)^a^	0	0	0
Robinson et al. 7	8	3 (37.5%)^b^	3 (37.5%)	2 (*NAB2ex7‐STAT6ex3*) (25%)	0
Mohajeri et al. 8	8	7 (87.5%)	0	0	1 (12.5%)
Barthelmeß et al. 9	27	25 (92.6%)	0	1 (*NAB2ex4‐STAT6ex18*) (3.7%)	1 (3.7%)
Vogels et al. 10	8	8 (100%)	0	0	0
Akaike et al. 11	24	18 (75%)	0	3 (*NAB2ex2‐STAT6ex6*) (12.5%)2 (*NAB2‐ex6‐STAT6ex18*) (8.3%)1(*NAB2ex2‐STAT6ex2*) (4.2%)	0
Current study	34	29 (85.3%)	5 (14.7%)	0	0
Total	114	95 (83%)	8 (7%)	9 (8%)	2 (2%)

a The original paper reported *NAB2* exon 4 fused to 5′‐UTR (5′‐untranslated region) of *STAT6*, which could be annotated to *NAB2ex4‐STAT6ex2* based on the different gene reference.

b Include a case with *NAB2ex4‐STAT6ex5* fusion.

All the NAB2‐STAT6 chimeric proteins have a truncated transcriptional repressor domain (RD) of NAB2 with an in‐frame fusion to the transcriptional activation domain (TAD) of STAT6 7. Because NAB2 acts a suppressing regulator for EGR1 transcription factor, a defective NAB2 fused with the TAD of STAT6 leads to constitutive activation of EGR‐mediated transcription targets, notably *NAB2*,* NAB1*,* IGF2*,* EGF2*,* PDGFD*,* FGFR1,* and *NTRK1*. *NAB2*‐*STAT6* fusion is considered a tumor‐initiating event across benign and malignant SFTs. Other secondary genetic alterations may also play key roles in the tumor progression, such as *TERT* or *TP53* mutations 11. The *NAB2ex4‐STAT6ex2/3* chimeric protein presumably fuses the truncated RD of NAB2 and the coiled‐coil domain of STAT6, whereas the *NAB2ex6‐STAT6ex16/17* fusions result in truncated RD of NAB2 fused with Src homology of STAT6. Currently, there are no cell‐based data to compare the functional difference between various NAB2‐STAT6 fusion proteins.

As illustrated in our current study, SFTs may overlap with other spindle cell tumors in morphologic appearance. For an intrathoracic location, sarcomatoid lung carcinoma and mesothelioma are two mimics that must be kept in mind in a differential diagnosis. Soon after the identification of *NAB2*‐*STAT6* fusion, the great majority of SFTs were found to exhibit diffuse and strong STAT6 nuclear staining, irrespective of the anatomic location and benign versus malignant histomorphology. This characteristic feature has made STAT6 immunohistochemistry emerge as a robust diagnostic of SFTs, especially in CD34‐negative cases 12, 13. Even in fusion‐negative SFTs, STAT6 nuclear immunoreactivity has a superb diagnostic value 9, 10. STAT6 is a transcription factor of the STAT family that is shuttled between the cytoplasm and nucleus and binds to specific DNA promoters upon relocation into the nucleus 22. The nuclear entry of STAT6 in SFTs is thought to result from the *NAB2*‐*STAT6* fusion containing the TAD of STAT6 that drives the neoplastic transformation. Although no STAT inhibitor is approved for clinical use to date, the constitutively activated STAT pathway in SFTs may emerge as a promising therapeutic target for inoperable or refractory atypical or malignant SFTs. In this regard, direct inhibition of STAT3 has been shown to reduce tumor growth and prolong survival in both animal models and human studies. There is an ongoing development of structurally analogous compounds in many laboratories and pharmaceutical companies 23, 24.

In conclusion, we demonstrated the prognostic significance of histological classification and the diagnostic utility of STAT6 immunohistochemistry in 52 intrathoracic SFTs. In addition, the integrative compilation of data regarding *NAB2‐STAT6* gene fusion in our study and previous series clearly indicates the predominance of the *NAB2ex4‐STAT6ex2/3* fusion variant in intrathoracic SFTs. Because some controversies about the associations of different fusion variants with clinicopathological features or prognostic implications are not fully clarified, future large‐scale studies coupled with a functional decipherment of different fusion variants are needed.

## Conflict of Interest

The authors declare no conflicts of interest.

## Supporting information


**Figure S1.** Diagnostic distinction and absence of STAT6 nuclear staining in thoracic histological mimics of solitary fibrous tumors.

**Data S1.** Materials and methods.
**Table S1.** Primer sets and products obtained in RT‐PCR assays.
