# Supplementary figures and images for: The clinicopathological significance of NAB2‐STAT6 gene fusions in 52 cases of intrathoracic solitary fibrous tumors
Source: Cancer Med. 2015 Dec 21;5(2):159–68. doi: 10.1002/cam4.572 (PMC4735766; doi:10.1002/cam4.572)

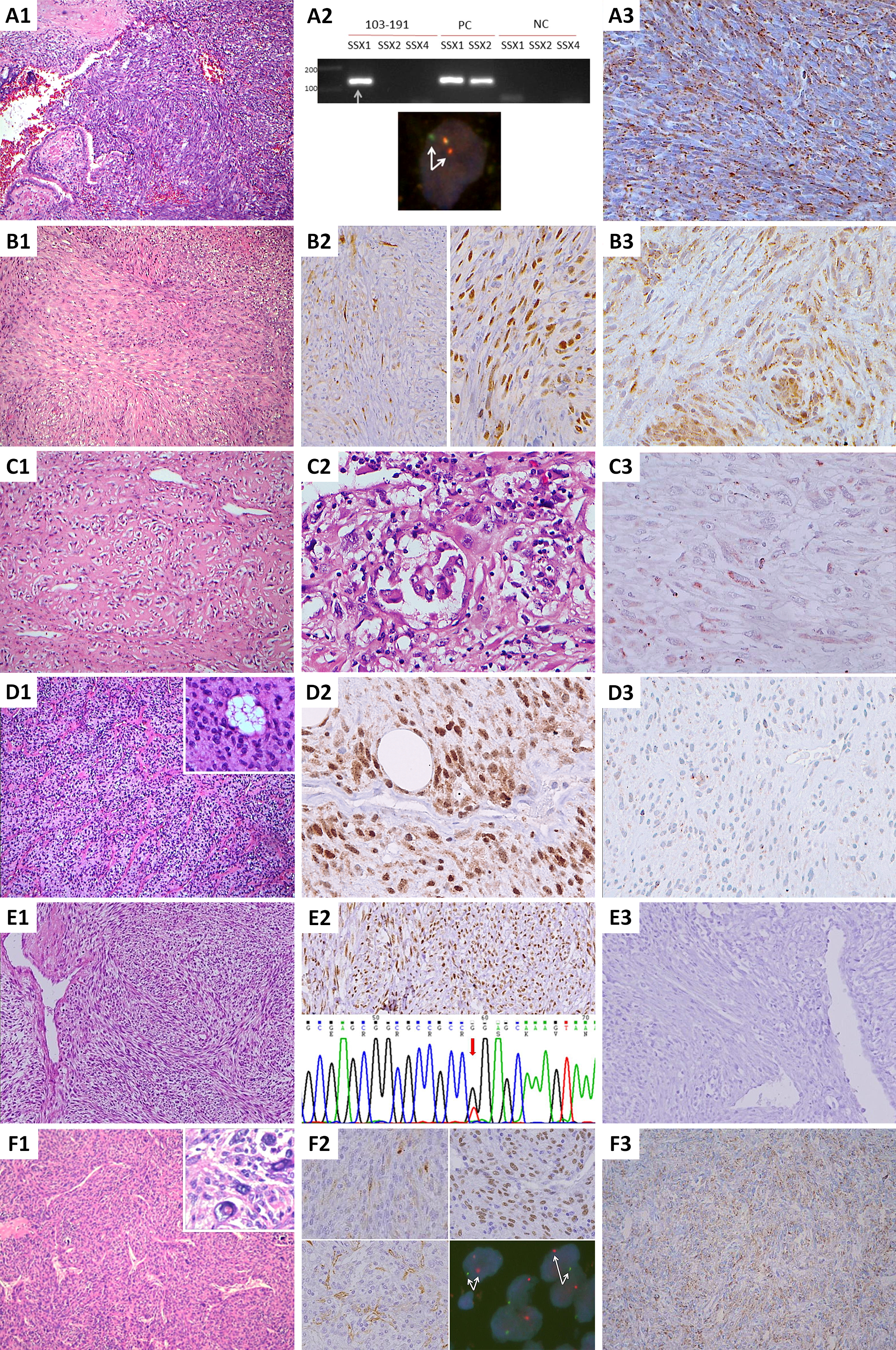

Supplement: Supplementary file 1 — Figure S1. Diagnostic distinction and absence of STAT6 nuclear staining in thoracic histological mimics of solitary fibrous tumors. [file CAM4-5-159-s001.tif]
