# Supplementary material for: The clinicopathological significance of NAB2‐STAT6 gene fusions in 52 cases of intrathoracic solitary fibrous tumors
Source: Cancer Med. 2015 Dec 21;5(2):159–68. doi: 10.1002/cam4.572 (PMC4735766; doi:10.1002/cam4.572)
Supplement: Supplementary file 2 — Data S1. Materials and methods. Table S1. Primer sets and products obtained in RT‐PCR assays. [file CAM4-5-159-s002.doc]

**Supplemental Materials and Methods**

Using the same antigen retrieval method and detection kit for immunohistochemistry, we diagnosed a pulmonary PEComa with the antibodies directed against smooth muscle actin (1:1000, 1A4, Thermo scientific), HMB-45 (1:50, HMB45, DAKO), Melan-A (1:200, A103, Thermo scientific), and TFE3 (1:1500, SC-5958, Santa Cruz).

The RT-PCR assays for *SS18-SSX1* and *SS18-SSX2* gene fusions and break-part FISH targeting *SYT* diagnostic of primary monophasic synovial sarcomas were performed as previously described.^1^ The *MYOD1* pL122R point mutation characteristic of spindle/sclerosing rhabdomyosarcomas was detected by Saner sequencing of PCR-amplified DNA materials extracted from formalin-fixed, paraffin-embedded (FFPE) tissues. Primers, *MYOD1*-for: CCTACTGTGGGCCTGCAAG and *MYOD1*-rev: GGATCTCCACCTTGGGCAAC, were used to perform PCR targeting *MYOD1*, with the product size being 170 bp and annealing temperature set at 60°C. To confirm the *TEF3* gene rearrangement in the pulmonary spindle PEComa, a commercial break-apart FISH probe targeting the *TFE3* gene (Z-2109-200, Zytovision) was applied on FFPE tissue sections of 4-μm thickness according to the manufacture’s protocol with provided kit. The sections was counterstained with DAPI II in mounting medium and scored by evaluating at least non-overlapping 100 tumoral nuclei. Positive cases were defined as those having split red and green signals separated by a distance at least twice the signal diameter in at least 20 of 100 counted tumoral nuclei.

Reference:

1. Amary MF, Berisha F, Bernardi Fdel C, et al. Detection of SS18-SSX fusion transcripts in formalin-fixed paraffin-embedded neoplasms: analysis of conventional RT-PCR, qRT-PCR and dual color FISH as diagnostic tools for synovial sarcoma. *Mod Pathol* 2007;20:482-96.

**Supplemental Table-1. Primer sets and products obtained in RT-PCR assays**

|  | Primer pair | Sequence | Fusion type obtained (*NAB2* exon - *STAT6* exon) | Size of products |
| --- | --- | --- | --- | --- |
| I | *NAB2* exon 3 forward  *STAT6* exon 3 reverse | 5’- CCCGAGAGAGCACCTACTTG  5’- GGTGCTGGACAGTGTCTGAA | 4-2  4-*NAB2* intron 4-3^#^ | 303bp  153bp |
| II | *NAB2* exon 6 forward-1  *STAT6* exon 17 reverse | 5’- GCAGACACTGATGGACGAG  5’- TGGGCTTCTTGGGATAGAGA | 6-16 | 219bp |
| III | *NAB2* exon 6 forward-2  *STAT6* exon 18 reverse | 5’- ACATCCTGCAGCAGACACTG  5’- TCTGGGGTAGGAAGTGGTTG | 6-17  6-*STAT6* intron 16-17 | 199bp 217bp |

^#^, breakpoints within exons

**Supplemental Figure 1. Diagnostic distinction and absence of STAT6 nuclear staining in thoracic histological mimics of solitary fibrous tumors.** A representative primary pulmonary monophasic synovial sarcoma (***A1,*** 100X) beneath the bronchial mucosa exhibited fascicular proliferation of hyperchromatic spindle cells with overlapping atypical nuclei, as confirmed by *SS18-SSX1* gene fusion (arrow) using RT-PCR (***A2,*** *upper*) and *SYT* gene rearrangement (split arrows) using break-apart FISH (***A2,*** *lower*). No nuclear expression of STAT6 was observed in this synovial sarcoma, except for some non-specific granular staining in the cytoplasm (***A3***). In sarcomatoid mesotheliomas (***B1*,** 100X) and sarcomatoid carcinomas (***C1***, 100X), the diagnosis of one each representative case was supported by the reactivity to both calretinin (***B2,*** *left*) and WT1 (***B2,*** *right*) for the former and by the presence of abortive papillary glands for the latter (***C2***). Neither sarcomatoid mesotheliomas (***B3***) nor sarcomatoid carcinomas (***C3***) showed STAT6 nuclear expression. One primary mediastinal dedifferentiated liposarcoma (***D1,*** 100X) showed increased cellularity of moderately pleomorphic cells set in a fibromyxoid stroma with prominent vascular network and occasional lipoblasts (***D1,*** *inset*) and CDK4 nuclear overexpression characteristic of this tumor type (***D2***), while there was no nuclear labeling of STAT6 (***D3***). One mediastinal spindle cell/sclerosing rhabdomyosarcoma peculiarly displayed intersecting bundles of elongated spindly cells with interspersed staghorn vasculature on the left (***E1,*** 100X) and exhibited the typically diffuse MyoD1 nuclear reactivity (***E2,*** *upper*) and pathogonmic *MYOD1* pL122R mutation (***E2,*** *lower*). However, no nuclear labeling of STAT6 was found, either (***E3***). One pulmonary PEComa was composed of spindle to polygonal tumor cells growing around prominent thin-walled staghorn vessels (***F1,*** 100X) with eosinophilic granular cytoplasm and nuclear pseudoinclusion (***F1,*** inset) and diagnosed by focal expression of Melan-A (***F2****, left upper*), smooth muscle actin (***F2****, left lower*), strong TFE3 nuclear expression (***F2****, right upper*), and split red and green signals indicative of rearranged *TFE3* gene (split white arrows) by FISH (***F2****, right lower*). This *TFE3*-rearranged PEComa was also negative for STAT6 nuclear reactivity but showed non-specific cytoplasmic staining (***F3***).
